# Carbon and energy metabolism for the mixotrophic culture of *Chlorella vulgaris* using sodium acetate as a carbon source

**DOI:** 10.3389/fmicb.2024.1436264

**Published:** 2024-10-23

**Authors:** Xi Yan, Shengzhou Shan, Xiaohui Li, Qingshan Xu, Xiaojun Yan, Roger Ruan, Pengfei Cheng

**Affiliations:** ^1^College of Food Science and Engineering, Ningbo University, Ningbo, Zhejiang, China; ^2^Lijiang Cheng Hai Bao Er Biological Development Co., Ltd., Lijiang, Yunnan, China; ^3^School of Marine Sciences, Ningbo University, Ningbo, Zhejiang, China; ^4^Center for Biorefining, and Department of Bioproducts and Biosystems Engineering, University of Minnesota-Twin Cities, Saint Paul, MN, United States

**Keywords:** *Chlorella vulgaris*, mixotrophic culture, sodium acetate, carbon and energy metabolism, metabolic network

## Abstract

There has been an emergence of a diversity of microalgal mixotrophic synergistic mechanisms due to substrate differences. In this study, the effects of the mixotrophic culture of *Chlorella vulgaris* were examined. The maximum values of cell density, specific growth rate, and cell dry weight of *Chlorella vulgaris* were 3.52*10^7^ cells/mL, 0.75 d^−1^, and 3.48 g/L in the mixotrophic mode, respectively. These were higher than the corresponding values of photoautotrophic or heterotrophic modes. Moreover, it was found that the concentrations of sodium bicarbonate consumed by the *Chlorella vulgaris* under mixotrophic and photoautotrophic modes were 635 mg/L/d and 505 mg/L/d, respectively; the concentrations of sodium acetate consumed by the *Chlorella vulgaris* under mixotrophic and heterotrophic modes were 614 mg/L/d and 645 mg/L/d, respectively. The activity of Rubisco was 9.36 U/mL in the mixotrophic culture, which was 3.09 and 4.85 times higher than that of the photoautotrophic and heterotrophic modes, respectively. This indicated that the differences for the carbon source absorption efficiency of *Chlorella vulgaris* in the mixotrophy led to different internal metabolic efficiencies when compared to photoautotroph or heterotrophy. Additionally, *Chlorella vulgaris* exhibits a more rapid energy metabolism efficiency when operating in the mixotrophic mode.

## 1 Introduction

Microalgae are a group of microscopic, photosynthesizing, unicellular, or simple multicellular organisms (Wang et al., 2008). Microalgae, as carriers of soluble dietary fiber, are widely used in the production for important high-value-added products and considered to be an important source of healthy food for humans (de Jesus Raposo et al., 2016). Moreover, microalgae are rich in phenolic substances, vitamins, unsaturated fatty acids, and other biologically active substances, and can also be used as animal feed, and therefore known as the ‘superfood of the future’ (Wikfors and Ohno, 2001; Zhou et al., 2022). In addition, microalgae can fix CO_2_, nitrogen, and phosphorus pollutants and turn them into energy substances, thus reducing the ‘greenhouse effect’ and saving energy (Zhuang et al., 2023). Microalgae play an important role in various fields, but several factors, such as the high requirements of microalgae for the growth environment, the metabolic efficiency of active substances, and the low density of cell culture, have hindered the large-scale production of microalgal biomass (Ennaceri et al., 2023).

Studies have shown that the biomass of microalgae can be enhanced by improving the culture modes (Zhuang et al., 2018). The artificial cultivation process gives nutrients, light, and temperature, which in turn can promote the growth and metabolism of microalgae. In general, the main modes of microalgae culture are photoautotrophic, heterotrophic, and mixotrophic (Wang et al., 2014). Photoautotrophic mode is the use of photosynthesis by microalgae to convert light energy into cellular material. This culture process can be simple, and low-cost. However, due to a series of reasons such as a single energy source and the limitation of light, most microalgae are in a low state of cell density in the photoautotrophic culture (Zhang et al., 2011). Heterotrophic mode is a culture in which microalgae absorb external organic carbon only in dark conditions. Heterotrophic culture is not limited by light and can effectively increase biomass growth of microalgae (Tian-Yuan et al., 2014). However, the proportion of heterotrophic microalgae is very small, with high carbon source costs, and low carbon source utilization rates. Mixotrophic growth of microalgae combines both photoautotrophic and heterotrophic growth; so it can utilize both light energy and external inorganic carbon to provide CO_2_ for photosynthesis, as well as absorb external organic carbon for growth (Chen et al., 2023). Compared to photoautotrophic mode or heterotrophic mode, mixotrophic culture combines the advantages of photoautotrophic and heterotrophic mode and has the potential to produce higher biomass production (Shan et al., 2023a).

Currently, there is a diversity of research on microalgal mixotrophic culture mechanisms. Studies have suggested that the effect of mixotrophic culture is a sum of photoautotrophic and heterotrophic cultures (Lu et al., 2023). Moreover, it has also been found that the biomass of some microalgae in the mixotrophic mode may be higher than the sum of the photoautotrophic and heterotrophic modes (Shan et al., 2023b). This implies that there may be a synergistic effect of photoautotrophic and heterotrophic modes, under mixotrophic mode. Through this synergistic effect, the rate of carbon metabolism and energy metabolism of microalgae can be enhanced, which ultimately increases biomass production.

Previous studies have demonstrated that when glucose was utilized as an organic carbon source for *Chromochloris zofingiensis* in a mixotrophic culture mode, the up-regulation of the EMP pathway inhibits the Calvin-Benson-Bassham cycle (CBB; Zhang et al., 2021). Building on these findings, this study aims to identify an organic carbon source that does not excessively enhance the EMP pathway. In this research, sodium acetate was selected to investigate whether the synergistic mechanisms observed with glucose as the organic carbon source would be replicated. So, the metabolic mechanisms of *Chlorella vulgaris* in the mixotrophic mode, with sodium acetate as the organic carbon source, have been studied further. The physiological characteristics of *Chlorella vulgaris* in the mixotrophic mode were compared with those in photoautotrophic and heterotrophic modes. The rate of exogenous organic and inorganic carbon consumption was used as the entry point, to study the differences in the rate of carbon consumption of *Chlorella vulgaris* in the mixotrophic mode. The regulation of the specific pathway of carbon consumption was explored by activating key enzymes of the carbon metabolism process, which was to reveal the advantages of carbon metabolism in the mixotrophic mode.

## 2 Materials and methods

### 2.1 Microalgae strains and culture conditions

The microalgae strain used in this study was *Chlorella vulgaris*; it was provided by Microalgae Biotechnology Laboratory, Ningbo University, Ningbo, China. In this study, f/2 medium was used as the basic culture condition (Lananan et al., 2013). The microalgae were incubated on a shaker at 150 rpm. The incubation temperature was 25 ± 1°C; the light intensity was 5,000 ± 100 lux; the photoperiod was 12: 12 h (light: dark). The seawater and medium were sterilized at 121°C for 20 min and then cooled. The microalgal seed solution was diluted to 3 × 10^5^ cells/mL with seawater. The experimental groups were set up in photoautotrophic mode (NaHCO_3_), heterotrophic mode (sodium acetate), and mixotrophic mode (NaHCO_3_ + sodium acetate). Each flask is sealed with plastic film at the mouth and wrapped in 4 layers of gauze to eliminate contact with external air.

### 2.2 Microalgae cell number, growth rate, and dry weight measurements

1 mL of algae culture solution was taken and 100 *μ*L of Lugol’s iodine solution was added and shaken well to stain the fixed cells and counted every 48 h, using a hemocyte counting plate. The number of microalgae cells was observed in a counting plate grid. The counting formula was as follows:

Number of cells (cells/mL) = (80 grid cells/80) × 400 × 10^4^.

The specific growth rate (μ) of *Chlorella vulgaris* was calculated as follows:

μ = (ln Nt_1_-lnNt_0_)/(t_1_-t_0_).

μ—specific growth rate per day;

Nt_1_-cell density at specific incubation time t_1;_

Nt_0_—cell density at specific culture time t_0;_

t_1_—d_1_ corresponds to the culture time;

t_0_—d_0_-corresponding incubation time.

10 mL of microalgae culture was weighed every 48 h, and the microalgae cells were separated by centrifugation and stored in a light-protected solution of 0.2% glutaraldehyde. After cultivation, microalgal cells were dried to constant weight by centrifugation to determine their dry weights (Shan et al., 2023b).

### 2.3 Measurement of bicarbonate and acetate concentrations

The concentration of HCO_3_^-^ was determined by neutralization titration (Zhang et al., 2017). The concentration of acetate ions was determined using gas chromatography (Wittmann et al., 2000).

### 2.4 Measurement of enzyme activity

In this study, enzyme-linked immunosorbent assay (ELISA) was used for sequential determination of different enzymes. The enzyme activities measured included ribulose-1,5-bisphosphate carboxylase/oxygenase (Rubisco), citrate synthase (CS), fructose-1,6-bisphosphate aldolase (FBA), glyceraldehyde-3-phosphate dehydrogenase (GAPDH) and glucose-6-phosphate dehydrogenase (G6PDH). 50 mL of algae was taken in the stable stage, and centrifuged at 8000 r/min for 15 min, the supernatant was removed and transferred to a 2 mL homogenizing tube at 9000 r/min for 12 min. Then the supernatant was removed and homogenized with grinding beads, and the collected samples were centrifuged to obtain the supernatant. The double antibody one-step sandwich enzyme-linked immunosorbent assay was further used, and then the absorbance was measured at 450 nm with an enzyme-labeled instrument, and the standard curve was developed (Shan et al., 2023b).

### 2.5 Determination of chlorophyll fluorescence parameters

A chlorophyll fluorometer WATER-PAM (EDEE0228, Heinz Walz GmbH) was used to determine chlorophyll fluorescence parameters. Every 48 h, 1 mL of algal liquid was taken from each sample group and put into the tube. The tube was wrapped with tin foil and kept away from light for 15 min. Then, the algal liquid was poured into the specific test tube of the instrument under dark conditions and waited for the instrument to show FV/FM, Y(NO), Y(NPQ), and Y(II).

### 2.6 Determination of starch content

Starch content was determined using the Plant Starch Content Determination Kit (Nanjing Jianjian Bioengineering Institute). Separation of soluble sugar and starch in the sample was achieved using 80% ethanol; then the starch decomposed into glucose by acid hydrolysis, and the glucose was quantified by anthrone sulfate colorimetry to calculate the starch content (Lu et al., 2023).

### 2.7 Determination of lipid content

The chloroform-methanol method was used to determine the lipid concentration in the microalgal cells (Lam and Proctor, 2001). During the stabilization period of the microalgal cells, 50 mL (V1) of microalgal liquid was weighed and centrifuged. Methanol and chloroform were then added to the microalgae solution, and the extraction was repeated 2 times. The mixture was then centrifuged and transferred to a new glass tube (mg, W1). Then 61°C nitrogen gas was blown across the tube for 10 min to evaporate the chloroform. The sample was baked at 105°C for 3 h, cooled, and weighed as W2 (mg). The lipid content was then calculated:

Lipid content (%) = (W2–W1)/DW_V1_ × 100%.

### 2.8 Determination of protein content

Protein content was determined using the Coomassie brilliant blue method (Matejovicova et al., 1997). At the stable stage, 50 mL of algal liquid was weighed and the sample was prepared by centrifugation, grinding, and standing. Then 5 mL of Coomasil bright blue reagent was added, fully shaken and mixed, and left for 2 min. The absorbance value was determined at the wavelength of 595 nm using an enzyme marker and a standard curve was developed.

### 2.9 Statistical analysis

The experiments consisted of three parallel groups, and the mean of three independent replicates was calculated. Differences between the groups were analyzed using SPSS 20 software, with statistical significance defined as *p* < 0.05.

## 3 Results and discussion

### 3.1 Mixotrophic growth of *Chlorella vulgaris*

**Figure 1:**
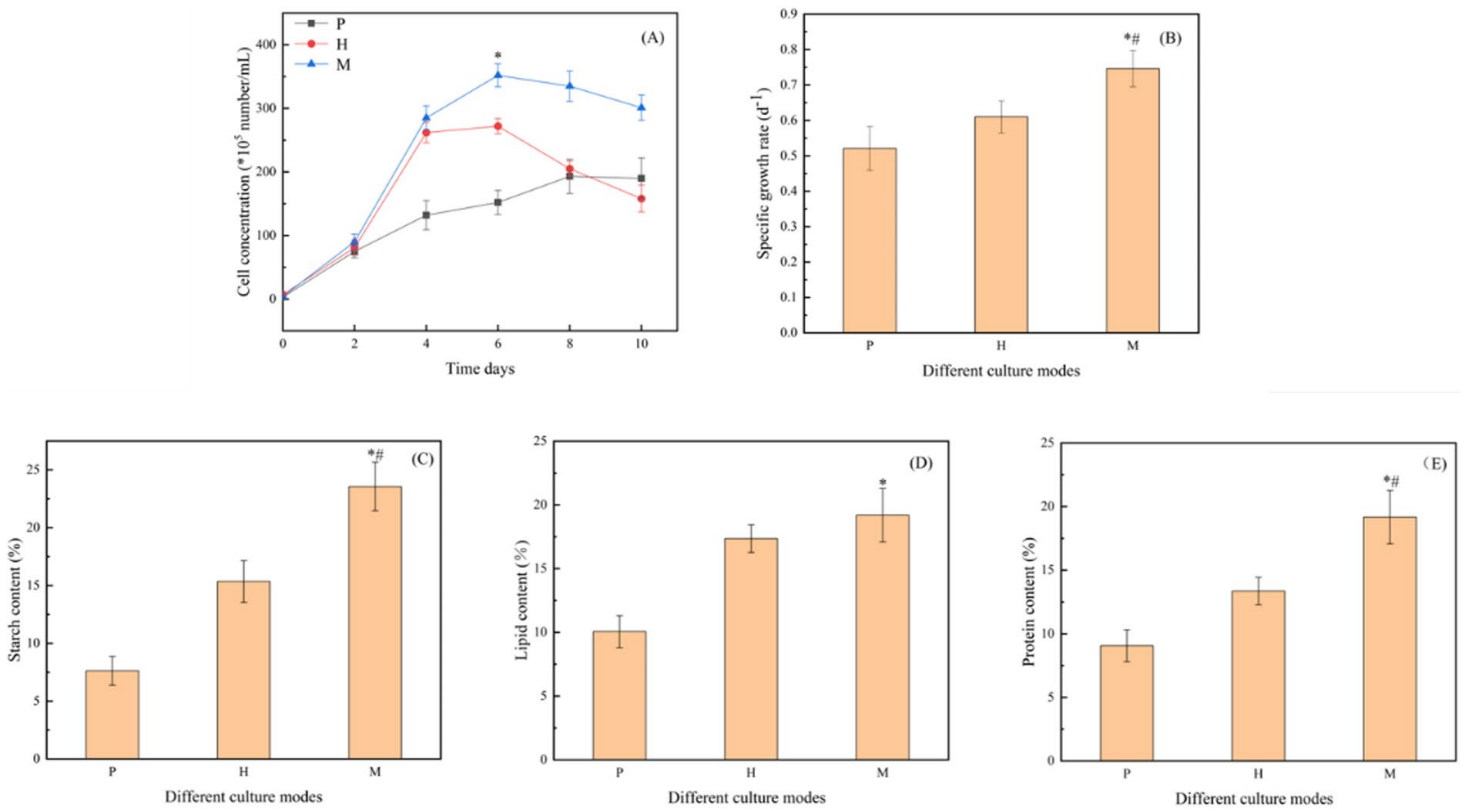
**(A)/(B)** Cell concentration/specific growth rate of *Chlorella* sp. under different culture modes; **(C–E)** Starch, lipid, and protein contents of *Chlorella* sp. under different culture modes. “P” represents photoautotrophic mode (3 g/L NaHCO_3_); “H” indicates heterotrophic mode (3 g/L sodium acetate); “M” indicates the mixotrophic culture mode (3 g/L NaHCO_3_ + 3 g/L sodium acetate); “*” indicates that there is a significant difference between “P” and “M,” and “#” indicates that there is a significant difference between “H” and “M” (*p* < 0.05).

Compared to photoautotrophic and heterotrophic modes, some species of microalgae have higher biomass and cell density when grown in the mixotrophic mode. In this study, the feasibility of the mixotrophic culture of *Chlorella vulgaris* was investigated. The mixotrophic culture modes included photoautotrophic (P) growth conditions, formed by the addition of sodium bicarbonate (3 g/L) and given a light intensity of 5,000 lux; heterotrophic (H) growth utilized the addition of sodium acetate (3 g/L) and avoided the use of light; mixotrophic (M) growth utilized the addition of both sodium bicarbonate (3 g/L) and sodium acetate (3 g/L) and also included light intensity of 5,000 lux. As shown in Figure 1A, compared to photoautotrophic and heterotrophic modes, *Chlorella vulgaris* had a significantly higher growth rate in the mixotrophic mode. The growth rate in the mixotrophic mode had a significant difference from day 6; the algal cell density of this strain reached a maximum of 3.52*10^7^ cells/mL on day 6, which was 2.32 and 1.29 times higher than that of the photoautotrophic and heterotrophic modes, respectively.

As shown in Figure 1B, the specific growth rates of *Chlorella vulgaris* were 0.52 d^−1^, 0.61 d^−1^, and 0.75 d^−1^ under photoautotrophic, heterotrophic, and mixotrophic modes, respectively. The specific growth rates under mixotrophic conditions reached 1.44 and 1.23 times those of photoautotrophic and heterotrophic, respectively. The data clearly show that compared to photoautotrophic and heterotrophic modes, the specific growth rates under mixotrophic conditions were significantly elevated. As shown in Table 1, the dry weights of *Chlorella vulgaris* under both heterotrophic and mixotrophic modes, up to the third day, were much higher than that of photoautotrophic. Meanwhile, the dry weights of *Chlorella vulgaris* in the mixotrophic mode were 2.76 g/L and 3.48 g/L on the fifth and eighth days, respectively, which were greater than the sum of photoautotrophic and heterotrophic modes.

**Table 1:** The dry weight of *Chlorella vulgaris* under different culture modes.

Culture modes DW(g/L)	Day3	Day5	Day8
P	0.48 ± 0.08	0.67 ± 0.14	0.74 ± 0.18
H	1.25 ± 0.21	1.94 ± 0.20	2.58 ± 0.36
M	1.28 ± 0.15^*^	2.76 ± 0.22^*#^	3.48 ± 0.30^*#^

“P” indicates photoautotrophic mode (3 g/L NaHCO_3_); “H” indicates heterotrophic mode (3 g/L sodium acetate); “M” indicates the mixotrophic mode (3 g/L NaHCO_3_ + 3 g/L sodium acetate). “*” indicates that there is a significant difference between “P” and “M”; and “#” indicates that there is a significant difference between “H” and “M”.

To further investigate the effect of the mixotrophic culture of *Chlorella vulgaris*, the starch, lipid, and protein contents of *Chlorella vulgaris* were measured in this study, up to the sixth day of culture. As shown in Figure 1C, the starch contents in photoautotrophic, heterotrophic, and mixotrophic modes were 7.62%, 15.35%, and 23.56%, respectively. The starch content in the mixotrophic mode was significantly higher than the other two cultivation modes, which reached 3.09 and 1.53 times of those of the photoautotrophic and heterotrophic modes, respectively. As shown in Figure 1D, the lipid content of 19.18% in the mixotrophic mode was higher than that of the other two culture modes, 1.9 and 1.1 times higher than that of the photoautotrophic and heterotrophic modes, respectively. It was shown that the protein content of 19.18% under mixotrophic mode was significantly higher than that of photoautotrophic and heterotrophic cultures, being 2.12 and 1.44 times higher than the corresponding values, respectively (Figure 1E).

Compared to photoautotrophic and heterotrophic modes, *Chlorella vulgaris* showed a significant increase in cell density and specific growth rate in the mixotrophic mode. At the same time, the starch, lipid, and protein contents also had significant advantages, and the dry weight of algal cells exceeded the sum of the photoautotrophic and heterotrophic modes. Therefore, *Chlorella vulgaris* has the advantage of being cultured in a mixotrophic culture.

The growth of *Chlorella vulgaris* is influenced by a variety of external environmental factors. Zheng et al. selected *Chlorella vulgaris* as the research object and found that the biomass of *Chlorella vulgaris* generally showed an upward trend with the increase of CO_2_ concentration (Zheng et al., 2021). The specific growth rate of *Chlorella vulgaris* was measured under different light intensities, and the results showed that the specific growth rate of *Chlorella vulgaris* increased, and then decreased, with the increase in light intensity (Yeh et al., 2010). Morais et al. used the type of carbon source (glycerol and glucose) as the experimental variables, and it was showed that the biomass production and lipid content of microalgae were best with the carbon source of 0.1 M glycerol (Morais et al., 2021). In other words, light and carbon sources are key factors affecting the growth and metabolism of microalgae, but their impact mechanisms may vary for different culture modes of microalgae.

### 3.2 Consumption of external carbon sources of *Chlorella vulgaris*

**Figure 2:**
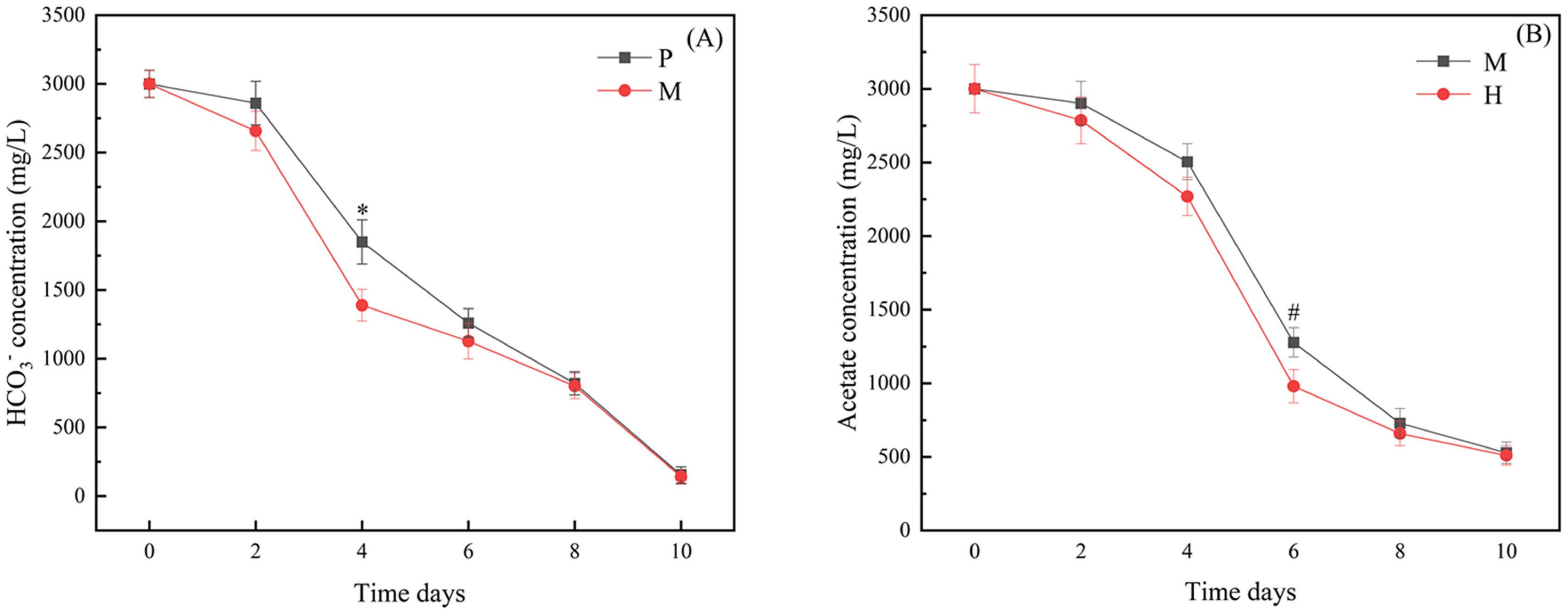
**(A)** The rate of bicarbonate consumption in photoautotrophic and mixotrophic modes; **(B)** The rate of acetate consumption in heterotrophic and mixotrophic modes. “*” indicates that there is a significant difference between “P” and “M,” and “#” indicates that there is a significant difference between “H” and “M”(*p* < 0.05).

The above studies showed that *Chlorella vulgaris* can utilize both bicarbonate and acetate for metabolic processes under the mixotrophic mode. The study of their metabolic interactions can help to reveal the mechanism that promotes rapid growth under the mixotrophic mode. Therefore, in this study, the consumption rates of carbonate and acetate ions were determined. As shown in Figure 2A, the overall bicarbonate consumption rate was higher in the mixotrophic mode than in the photoautotrophic mode. This effect was most obvious on days 2–6 and had a significant difference from day 2, with the bicarbonate consumed at a rate of about 635 mg/L/d, while the bicarbonate consumed in the photoautotrophic mode was at a rate of about 505 mg/L/d. As shown in Figure 2B, the overall acetate consumption rate was lower in the mixotrophic mode than in the heterotrophic mode, but significantly changed from day 4. The rate of acetate consumed in the mixotrophic mode was consumed at 614 mg/L/d, whereas the rate of acetate consumed in the heterotrophic mode was about 645 mg/L/d.

Notably, compared to the photoautotrophic mode, *Chlorella vulgaris* had a higher efficiency of external CO_2_ uptake in the mixotrophic mode. But, compared to the heterotrophic mode, *Chlorella vulgaris* had a reduced efficiency of external sodium acetate uptake. The CBB is the predominant pathway for CO_2_ assimilation in microalgae, from which *Chlorella vulgaris* produces substrates for a variety of metabolites dominated by glyceraldehyde 3-phosphate (G3P). Acetate ultimately supplies inorganic carbon and energy to the CBB through the tricarboxylic acid cycle (TCA). Therefore, the determination of the consumption rate of bicarbonate and acetate ions can provide a theoretical basis for studying the metabolic mechanisms in the mixotrophic mode.

### 3.3 Carbon metabolism of *Chlorella vulgaris*

**Figure 3:**
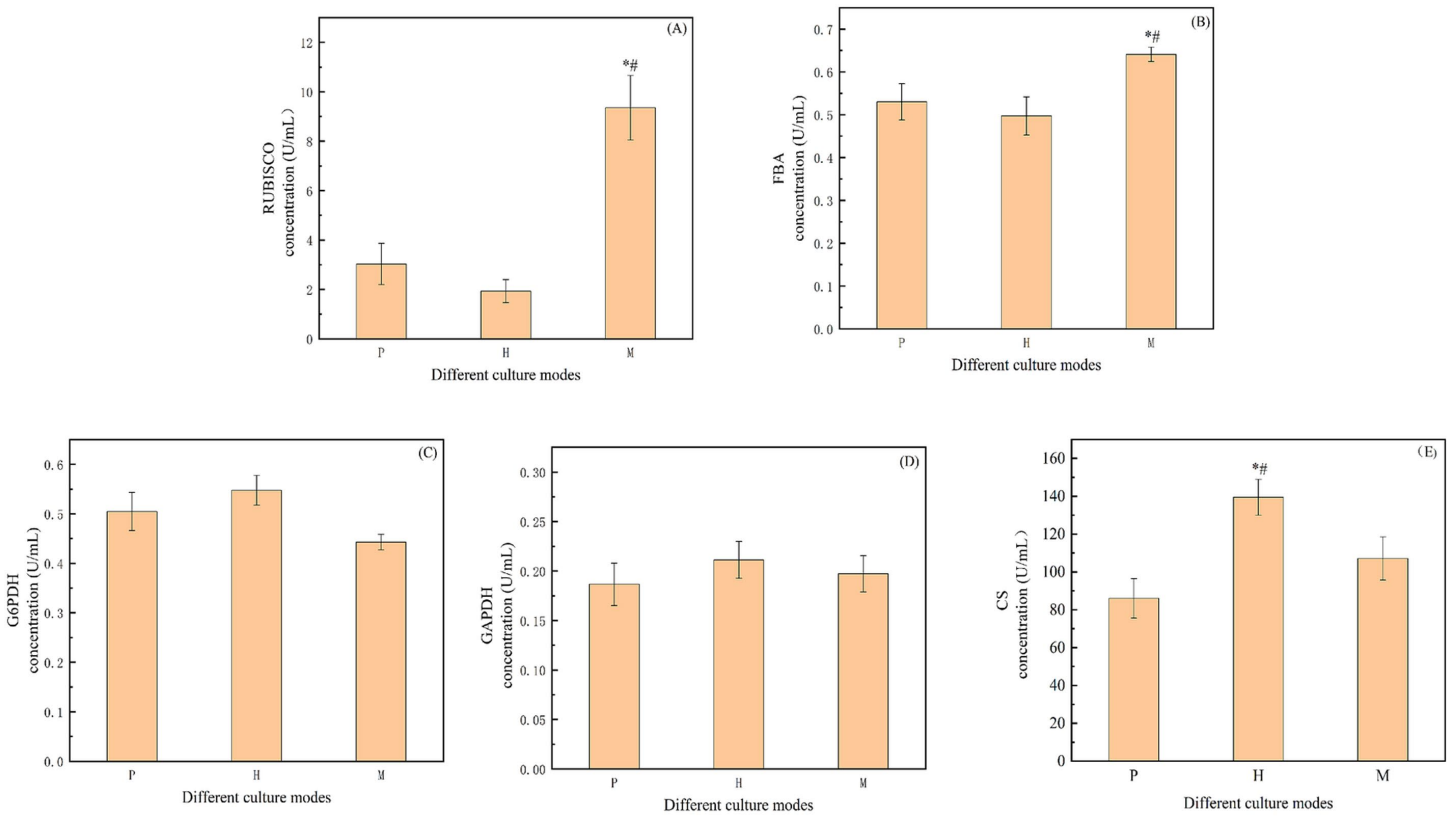
**(A)/(B)/(C)/(D)/(E)** The key enzyme activities in the EMP and TCA cycles. “RUBISCO”: 1, 5-diphosphate ribulose carboxylase/oxygenase; “FBA”: fructose-1, 6-diphosphate aldolase; “G6PDH”: glucose-6-phosphate dehydrogenase; “GAPDH”: glyceraldehyde 3-phosphate dehydrogenase; “CS”: Citrate synthase. “*” indicates that there is a significant difference between “P” and “M,” and “#” indicates that there is a significant difference between “H” and “M”(*p* < 0.05).

To investigate the mechanism of biomass output and related carbon source consumption of *Chlorella vulgaris* under the mixotrophic mode, key enzyme activities in related pathways were further determined in this study. CO_2_ uptake by microalgae is mainly from fixation by the CBB (Yin et al., 2022). In this study, it was determined the key enzymes in the CBB, are Rubisco and FBA. Rubisco is a key enzyme that determines the dark reaction rate in photosynthesis and participates in the dark reaction process of the photosynthetic reaction. It catalyzes the carboxylation of the substrate ribulose-1,5-bisphosphate (RuBP; McCully and McKinlay, 2016). Figure 3A shows that the Rubisco activity in the mixed mode was 9.36 U/mL, which was 3.09 and 4.85 times higher than that of the photoautotrophic and heterotrophic modes, respectively. It can be seen that the Rubisco enzyme activity in the mixotrophic was significantly higher than that in photoautotrophic or heterotrophic modes. FBA is involved in the third stage of the CBB. The RuBP, and plays an important role in the dark reaction stage of plant photosynthesis, fixing CO_2_ and converting 3C compounds to 6C compounds (Le Moigne et al., 2022). As shown in Figure 3B, the FBA activity in the mixotrophic culture was 0.64 U/mL, which was significantly higher than that in photoautotrophic and heterotrophic modes, and 1.21 and 1.29 times higher than that in photoautotrophic and heterotrophic, respectively. Therefore, compared to photoautotrophic and heterotrophic modes, the addition of sodium acetate in a mixotrophic culture of *Chlorella vulgaris* can lead to the up-regulation of the CBB.

It has been shown that, compared to the photoautotrophic culture, the mixotrophic mode with *Chromochloris zofingiensis* will accelerate the glycolytic pathway (EMP) with the exogenous addition of glucose (Zhang et al., 2021). The metabolites enter into the chloroplasts through the chloroplast transporter, which feedback inhibits the carbon sequestration mechanism. Therefore, in the present study, *Chlorella vulgaris* accumulates metabolites such as starch and lipids through the CBB and further decomposes the accumulated products through EMP and *β*-oxidation. Therefore, the rate of endogenous accumulation of starch was analyzed in different culture modes. G6PDH and GAPDH are two important enzymes of the EMP. G6PDH is a key enzyme in the pentose phosphate pathway (PPP), which converts glucose-6-phosphate into 6-phosphogluconic acid and NADPH, participating in regulating and limiting reaction rates (Yoshida et al., 2019). As shown in Figure 3C, the levels of G6PDH were 0.50 U/mL, 0.55 U/mL and 0.44 U/mL in photoautotrophic, heterotrophic, and mixotrophic conditions, respectively, which did not show significant differences. GAPDH is the core enzyme that catalyzes the interconversion of G3P and 1,3-bisphosphoglycerate (1,3-BPG) in the EMP pathway (Qvit et al., 2016). As shown in Figure 3D, the levels of GAPDH under photoautotrophic, heterotrophic, and mixotrophic modes were 0.19 U/mL, 0.21 U/mL, and 0.20 U/mL, in that order, and also did not show any significant differences.

In the metabolic pathway of *Chlorella vulgaris*, sodium acetate is mainly generated through the glyoxylate cycle (GAC) to produce acetyl coenzyme A, which subsequently participates in the TCA catalyzed by CS, and ultimately converts the metabolites into energy, carbon dioxide, and water. The experimental data in Figure 3E shows the CS activity in the three culture modes, heterotrophic, mixotrophic, and photoautotrophic, in descending order. The CS activity in the mixotrophic mode was 107.12 U/mL, which was 1.25 times than that in photoautotrophic and was 0.77 times lower than that in heterotrophic, respectively. Compared to the photoautotrophic mode, TCA efficiency was accelerated in the mixotrophic mode. Compared to the heterotrophic mode, it was decreased in the mixotrophic mode.

The CBB is an important carbon sequestration and biomass accumulation mode for *Chlorella vulgaris*; it is influenced by several aspects. It has been shown that different circadian rhythms significantly affect the biomass production mechanism of the CBB in microalgae. Specifically, Shi et al. used *P. helgolandica* as a study subject and set the light–dark ratios to 12 h: 12 h and 6 h: 18 h, respectively, after the addition of glucose. That study observed a significant difference in the starch accumulation, up to a maximum of 0.40 g L^−1^ d^−1^, which was interpreted to mean that the former may indicate an increase in EMP in the absence of light, and the latter may indicate an increased demand for RuBP synthesis (Shi et al., 2022). Light intensity is also strongly associated with the CBB in *Chlorella vulgaris*. The intensity of light may affect the relative magnitude of the activity of key enzymes in the CBB, which in turn affects the biomass output of *Chlorella vulgaris*. For example, Xie et al. studied *Chlorella sorokiniana* and observed that the activity of a series of enzymes involved in the CBB in algal cells were higher in the strong light culture than in the weak light culture, when *Chlorella sorokiniana* was given the strong light condition and the weak light condition, respectively. Moreover, the levels of Rubisco, a key enzyme of the CBB, were significantly higher than those of the low-light group, suggesting that the growth and metabolic processes of *Chlorella vulgaris* were promoted by the strong-light incubation under mixotrophic conditions, to enhance the metabolic efficiency of the CBB (Xie et al., 2016). In addition, different temperatures may also promote or inhibit the CBB pathway in microalgae to varying degrees. Ueno et al. investigated the effect of temperature on the CBB in microalgae by measuring the synthesis rates of 3-phosphoglyceric acid(3GP), fructose-1,6-bisphosphate, and fructose-6-phosphate(F6P) at three different temperatures. They found that the synthesis rate of these three substances was higher than that of the other two temperatures at a mid-to-high temperature of 38°C, and showed the lowest rate at a low temperature of 20°C (Ueno et al., 2016). This study suggested that medium to high temperatures promoted the metabolic rate of the CBB, while low temperatures inhibited it. In summary, external conditions such as circadian rhythms, light intensity, and temperature all influence the CBB.

### 3.4 Energy metabolism of *Chlorella vulgaris*

**Figure 4:**
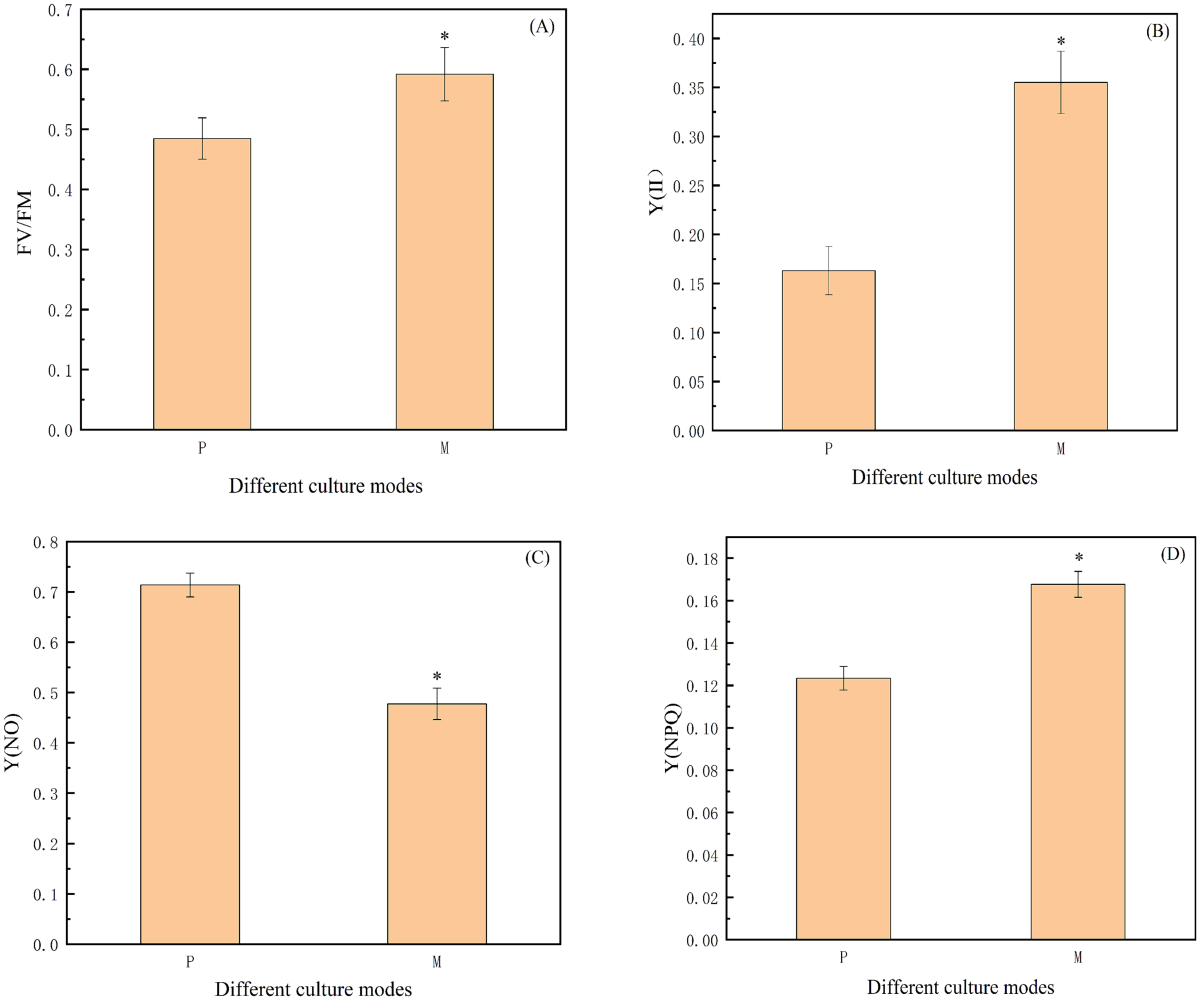
**(A-D)** Study on the photosynthetic parameters of *Chlorella vulgaris* under different culture modes. Note: “FV/FM” indicates the maximum photochemical quantum yield of PS II; “Y(II)” indicates the actual quantum yield; Y(NPQ) and Y(NO) represent the percentage of absorbed light energy consumed by non-photochemical quenching and chlorophyll fluorescence, respectively. “*” indicates that the difference between P and M is statistically significant (*p* < 0.05).

Chlorophyll fluorescence parameters are a set of variables, or constant values, used to describe the mechanism of plant photosynthesis and photosynthetic physiology. They are often regarded as intrinsic probes, to study the relationship between plant photosynthesis and the environment, and can be used as indicators of photosynthetic rate (Marxen et al., 2005). Therefore, this study used the chlorophyll fluorescence parameters to determine three modes, FV/FM, Y(II), Y(NO), and Y(NPQ). FV/FM represents the maximum photochemical quantum yield of PS II, which reflects the overall efficiency of microalgae photosynthesis as well as the efficiency of light energy utilization. *Chlorella vulgaris* is also more efficient at photosynthesis, and the corresponding increase in light energy utilization efficiency when the FV/FM ratio is higher. As shown in Figure 4A, the FV/FM ratio under photoautotrophic and mixotrophic modes were 0.48 and 0.59, respectively. Y(NPQ) and Y(NO) represent the percentage of absorbed light energy consumed by non-photochemical bursts and chlorophyll fluorescence, respectively. If the value of Y(NPQ) is too high, it means that this strain receives too much light, and if the value of Y(NO) is too high, it means that the photochemical energy conversion and the protective regulatory mechanisms (e.g., thermal dissipation) are not sufficient to consume the light energy completely. Y(II) then denotes the actual quantum yield (Meravi and Prajapati, 2020). The three chlorophyll fluorescence parameters, Y(II), Y(NO), and Y(NPQ), exist in the following relational equation: Y(II) + Y(NPQ) + Y(NO) = 1. Figure 4B shows that Y(II) under mixotrophic conditions was 0.36, which was 2.18 times higher than that of photoautotrophic culture. Figure 4C shows that Y(NO) was 0.71 and 0.48 under photoautotrophic and mixotrophic modes, respectively. Figure 4D shows that *Chlorella vulgaris* cultured in mixotrophic mode had a Y(NPQ) of 0.17, which was 1.36 times higher than that of photoautotrophic, and significantly higher than that of photoautotrophic.

Overall, compared to the photoautotrophic mode, *Chlorella vulgaris* has a better utilization of light energy in the mixotrophic mode. Under the mixotrophic mode, chloroplasts play an important coordinating role in energy and carbon metabolism. The mixotrophic mode allows the algal cells of *Chlorella vulgaris* to more fully utilize energy and organic carbon, than photoautotrophic or heterotrophic modes, thus affecting the accumulation of nutrients such as chlorophyll, lipids, and proteins in the algal cells of *Chlorella vulgaris*. Therefore, in the present study, chlorophyll fluorescence parameters of *Chlorella vulgaris* were determined to demonstrate that the rate of energy metabolism in the mixotrophic mode was faster than that of photoautotrophic and heterotrophic.

### 3.5 Carbon and energy metabolism in *Chlorella vulgaris* with acetic acid as an organic carbon source

Based on the above studies, the mixotrophic metabolism of *Chlorella vulgaris* when acetic acid was used as a culture condition can be summarized as follows: Compared to photoautotrophic and heterotrophic modes, the simultaneous addition of sodium bicarbonate and sodium acetate resulted in a more stable equilibrium of the CBB, TCA, EMP, GAC, and photoreaction phases in *Chlorella vulgaris* under the mixotrophic modes.

**Figure 5:**
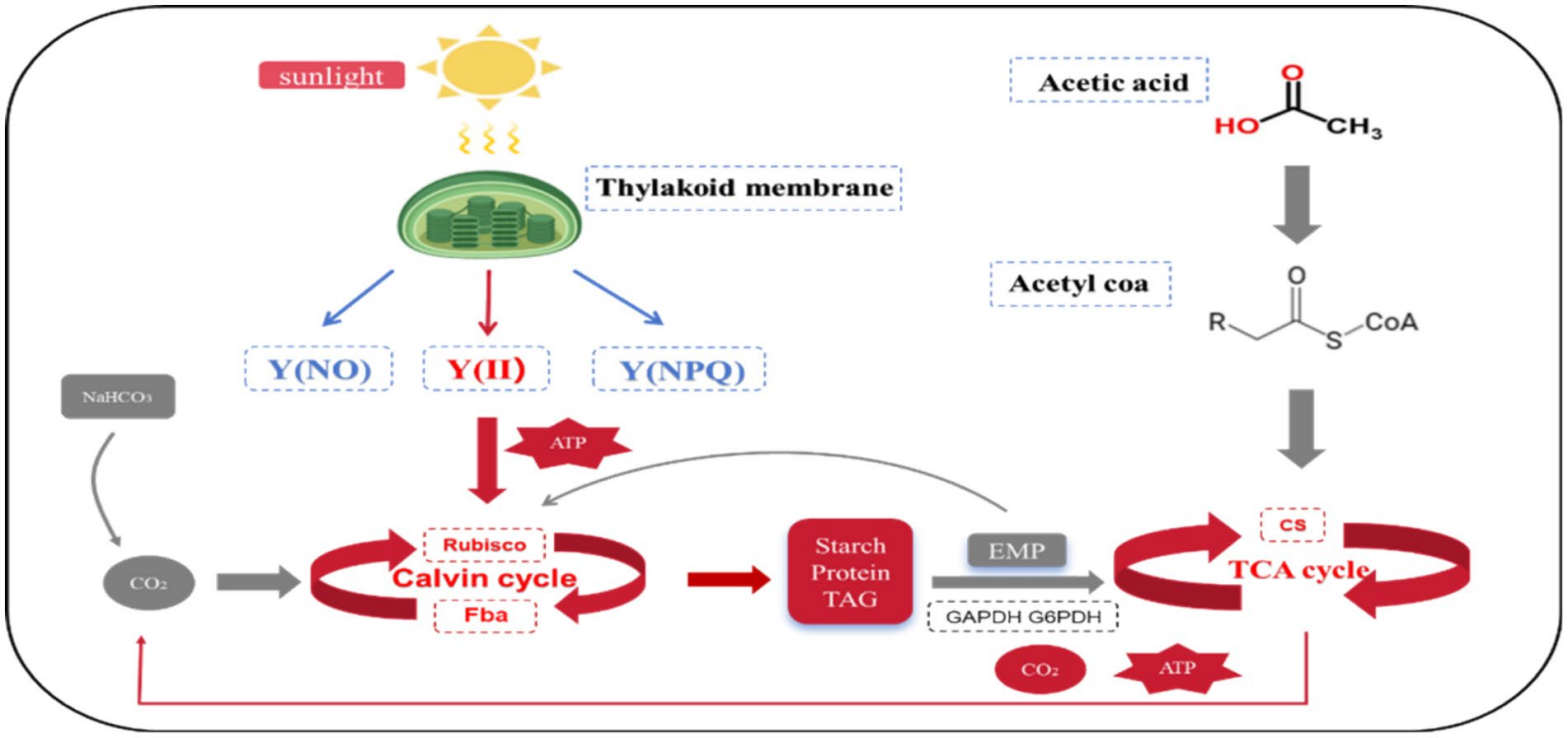
Carbon and energy metabolism of *Chlorella vulgaris* in mixotrophic mode. Red: Upregulated; Blue: Down. Enzyme: “Rubisco”: ribulose 1, 5-diphosphate carboxylase/oxygenase; “FBA”: fructose-1, 6-diphosphate aldolase; “GAPDH”: glyceraldehyde 3-phosphate dehydrogenase; “G6PDH”: glucose-6-phosphate dehydrogenase; “CS”: Citrate synthase. Chlorophyll fluorescence parameter: “Y(II)” represents the actual quantum yield; Y(NPQ) and Y(NO) represent the percentage of absorbed light energy consumed by non-photochemical quenching and chlorophyll fluorescence, respectively.

As shown in Figure 5, compared to photoautotrophic culture, *Chlorella vulgaris* in the mixotrophic mode resulted in an up-regulation of the CBB due to the incorporation of organic carbon and sodium acetate. When sodium acetate is employed as an organic carbon source, the efficiency of the EMP in *Chlorella vulgaris* is diminished. This hinders a sufficient quantity of corresponding cytoplasmic metabolites from entering the CBB in the cytoplasm, thereby making it impossible for Rubisco to be subjected to feedback inhibition by EMP. This further up-regulated the activity of Rubisco, explaining higher uptake efficiency and higher biomass output of external inorganic carbon. The addition of sodium acetate led to an overall upregulation of the TCA, producing more ATP and CO_2_, and may also serve as a reason for accelerating the metabolic efficiency of the CBB. Energy metabolism showed a significant up-regulation of FV/FM with Y(II) in the mixotrophic mode, along with an increase in the metabolic efficiency of the TCA, suggesting that the energy supply required for the up-regulation of the CBB comes from both cystoid and mitochondria. When sodium acetate is used as an organic carbon source, the efficiency of the CBB cycle metabolism in *Chlorella vulgaris* is not inhibited by cytoplasmic metabolites, which is different from when glucose is used. This suggests that the CO_2_ absorption efficiency of *Chlorella vulgaris* from the environment is promoted under these conditions. Consequently, this indicates that the synergistic mechanisms involved in *Chlorella vulgaris* metabolism vary depending on the organic carbon source utilized. This observation underscores the non-absoluteness of carbon metabolism mechanisms in microalgae, highlighting the adaptability and variability of their metabolic processes (Zhang et al., 2021).

Compared to the heterotrophic mode, *Chlorella vulgaris* in the mixotrophic mode resulted in an upregulation of the CBB due to the addition of inorganic carbon and sodium bicarbonate. The rate of endogenous amylase hydrolysis, which was slightly decreased in the mixotrophic mode, was not significantly different from the heterotrophic culture. Similarly, there would be no interference from the cytoplasm in the mixotrophic mode in the acceleration of the metabolic efficiency of the CBB by sodium bicarbonate. However, the addition of sodium bicarbonate reduced the demand of *Chlorella vulgaris* for sodium acetate in the mixotrophic mode, as compared to the heterotrophic mode. This resulted in a downgrading of the efficiency of the TCA, which explains the lower rate of consumption and higher biomass of the external organic carbon-sodium acetate. Energy metabolism shows that energy comes almost entirely from the mitochondria due to the absence of a photoreaction phase in the heterotrophic mode, explaining the elevated metabolic efficiency of *Chlorella vulgaris* of the TCA.

## 4 Conclusion

In this study, the metabolism of carbon and energy was utilized to explain the enhancement of biomass, and the reason for the change in the rate of consumption of carbon under the mixotrophic mode. Compared to the photoautotrophic mode, the addition of sodium bicarbonate to increase the biomass of *Chlorella vulgaris* in the mixotrophic mode, and the absence of significant up-regulation of the EMP with sodium acetate as the source of organic carbon, will not lead to an inhibitory effect on the activity of the carbon sequestering enzyme Rubisco. Thus, *Chlorella vulgaris* increases the efficiency of CO_2_ uptake. The energy required comes from both the photoreaction phase and the TCA. Down-regulation of TCA-related enzyme activity in *Chlorella vulgaris* in mixotrophic mode, compared to that in the heterotrophic mode, explained the decreased efficiency of sodium acetate uptake, and the energy was exclusively derived from the TCA.

## Data Availability

The raw data supporting the conclusions of this article will be made available by the authors, without undue reservation.
