# Correction: Carbon and energy metabolism for the mixotrophic culture of *Chlorella vulgaris* using sodium acetate as a carbon source

**DOI:** 10.3389/fmicb.2026.1889516

**Published:** 2026-06-22

**Authors:** Xi Yan, Shengzhou Shan, Xiaohui Li, Qingshan Xu, Xiaojun Yan, Roger Ruan, Pengfei Cheng

**Affiliations:** 1 College of Food Science and Engineering, Ningbo University, Ningbo, Zhejiang, China; 2 Lijiang Cheng Hai Bao Er Biological Development Co., Ltd., Lijiang, Yunnan, China; 3 School of Marine Sciences, Ningbo University, Ningbo, Zhejiang, China; 4 Center for Biorefining, and Department of Bioproducts and Biosystems Engineering, University of Minnesota-Twin Cities, Saint Paul, MN, United States

**Keywords:** *Chlorella vulgaris*, mixotrophic culture, sodium acetate, carbon and energy metabolism, metabolic network

There was a mistake in Figure 1 as published. The y-axis labels in Figure 1C and Figure 1E were incorrectly labelled as “Starch concentration (g/L)” and “Protein concentration (g/L)”. The correct labels should read as “Starch content (%)” and “Protein content (%).” The corrected Figure 1 and its caption appear below.

**Figure 1:**
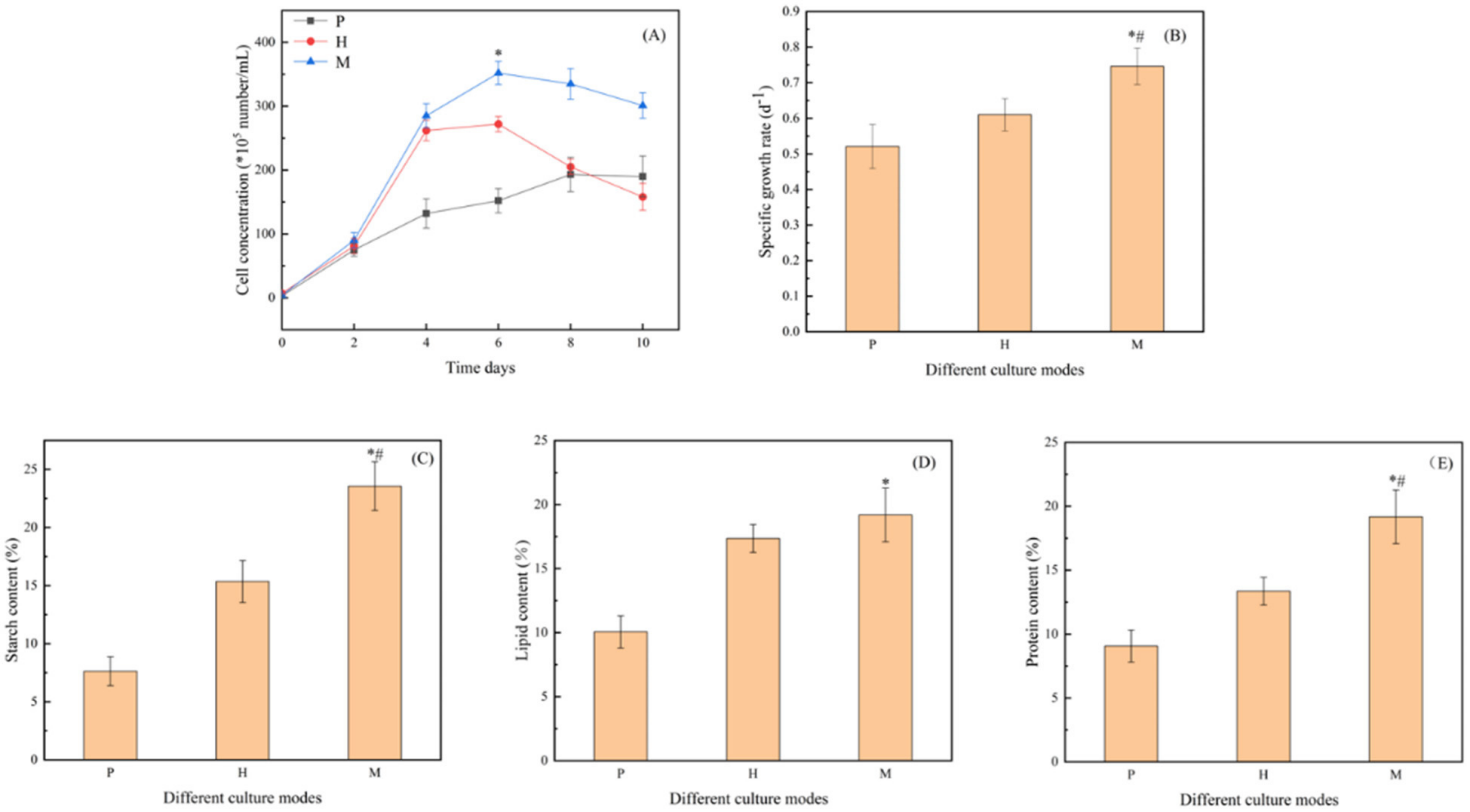
**(A)/(B)** Cell concentration/specific growth rate of Chlorella sp. under different culture modes; **(C–E)** Starch, lipid, and protein contents of Chlorella sp. under different culture modes. “P” represents photoautotrophic mode (3 g/L NaHCO_3_); “H” indicates heterotrophic mode (3 g/L sodium acetate); “M” indicates the mixotrophic culture mode (3 g/L NaHCO_3_ + 3 g/L sodium acetate); “*” indicates that there is a significant difference between “P” and “M,” and “#” indicates that there is a significant difference between “H” and “M” (*p* < 0.05).

A correction has been made to the section **Results and discussion**, subsection *3.1 Mixotrophic growth of Chlorella vulgaris*, Paragraph 3. The incorrect paragraph reads as, “To further investigate the effect of the mixotrophic culture of *Chlorella vulgaris*, the starch, lipid, and protein contents of *Chlorella vulgaris* were measured in this study, up to the sixth day of culture. As shown in Figure 1C, the starch contents in photoautotrophic, heterotrophic, and mixotrophic modes were 7.62 g/L, 15.35 g/L, and 23.56 g/L, respectively. The starch content in the mixotrophic mode was significantly higher than the other two cultivation modes, which reached 3.09 and 1.53 times of those of the photoautotrophic and heterotrophic modes, respectively. As shown in Figure 1D, the lipid content of 19.18 g/L in the mixotrophic mode was higher than that of the other two culture modes, 1.9 and 1.1 times higher than that of the photoautotrophic and heterotrophic modes, respectively. It was shown that the protein content of 19.18 g/L under mixotrophic mode was significantly higher than that of photoautotrophic and heterotrophic cultures, being 2.12 and 1.44 times higher than the corresponding values, respectively (Figure 1E).”

The corrected paragraph should read as, “To further investigate the effect of the mixotrophic culture of *Chlorella vulgaris*, the starch, lipid, and protein contents of *Chlorella vulgaris* were measured in this study, up to the sixth day of culture. As shown in Figure 1C, the starch contents in photoautotrophic, heterotrophic, and mixotrophic modes were 7.62%, 15.35%, and 23.56%, respectively. The starch content in the mixotrophic mode was significantly higher than the other two cultivation modes, which reached 3.09 and 1.53 times of those of the photoautotrophic and heterotrophic modes, respectively. As shown in Figure 1D, the lipid content of 19.18% in the mixotrophic mode was higher than that of the other two culture modes, 1.9 and 1.1 times higher than that of the photoautotrophic and heterotrophic modes, respectively. It was shown that the protein content of 19.18% under mixotrophic mode was significantly higher than that of photoautotrophic and heterotrophic cultures, being 2.12 and 1.44 times higher than the corresponding values, respectively (Figure 1E).”

The term “concentration” has been replaced with “content” in the Materials and methods' sub-section headings cited here: “2.6 Determination of starch concentration” has been corrected to “2.6 Determination of starch content”; “2.7 Determination of lipid concentration” has been corrected to “2.7 Determination of lipid content” “2.8; Determination of protein concentration” has been corrected to “2.8 Determination of protein content”.

A correction has been made to the equation given in Materials and methods, subsection 2.7 Determination of lipid content: “Lipid concentration (g/L) = (W2–W1)/V1.” Has been corrected to “Lipid content (%) = (W2–W1)/DW_V1_ × 100%.

The original version of this article has been updated.

